# Glucocorticoid-induced leucine zipper “quantifies” stressors and increases male susceptibility to PTSD

**DOI:** 10.1038/s41398-019-0509-3

**Published:** 2019-07-25

**Authors:** Maya A. Lebow, Mariana Schroeder, Michael Tsoory, Dorin Holzman-Karniel, Divya Mehta, Shifra Ben-Dor, Shosh Gil, Bekh Bradley, Alicia K. Smith, Tanja Jovanovic, Kerry J. Ressler, Elisabeth B. Binder, Alon Chen

**Affiliations:** 1 Department of Neurobiology, Weizmann Institute of Science, 76100 Rehovot, Israel; 2 Department of Stress Neurobiology and Neurogenetics, Max Planck Institute of Psychiatry, 80804 Munich, Germany; 3 Department of Veterinary Resources, Weizmann Institute of Science, 76100 Rehovot, Israel; 4 Department of Translational Psychiatry, Max Planck Institute of Psychiatry, 80804 Munich, Germany; 5 Department of Biological Services, Bioinformatics and Biological Computing Unit, Weizmann Institute of Science, 76100 Rehovot, Israel; 6 Atlanta Veterans Affairs Medical Center, Decatur, GA USA; 7 Department of Psychiatry and Behavioral Sciences, Emory University School of Medicine, Atlanta, GA 30322 USA

**Keywords:** Molecular neuroscience, Psychiatric disorders

## Abstract

Post-traumatic stress disorder (PTSD) selectively develops in some individuals exposed to a traumatic event. Genetic and epigenetic changes in glucocorticoid pathway sensitivity may be essential for understanding individual susceptibility to PTSD. This study focuses on PTSD markers in the glucocorticoid pathway, spotlighting glucocorticoid-induced leucine zipper (GILZ), a transcription factor encoded by the gene *Tsc22d3* on the X chromosome. We propose that GILZ uniquely “quantifies” exposure to stressors experienced from late gestation to adulthood and that low levels of GILZ predispose individuals to PTSD in males only. GILZ mRNA and methylation were measured in 396 male and female human blood samples from the Grady Trauma Project cohort (exposed to multiple traumatic events). In mice, changes in glucocorticoid pathway genes were assessed following exposure to stressors at distinct time points: (i) CRF-induced prenatal stress (_CRF-induced_PNS) with, or without, additional exposure to (ii) PTSD induction protocol in adulthood, which induces PTSD-like behaviors in a subset of mice. In humans, the number of traumatic events correlated negatively with GILZ mRNA levels and positively with % methylation of GILZ in males only. In male mice, we observed a threefold increase in the number of offspring exhibiting PTSD-like behaviors in those exposed to both _CRF-induced_PNS and PTSD induction. This susceptibility was associated with reduced GILZ mRNA levels and epigenetic changes, not found in females. Furthermore, virus-mediated shRNA knockdown of amygdalar GILZ increased susceptibility to PTSD. Mouse and human data confirm that dramatic alterations in GILZ occur in those exposed to a stressor in early life, adulthood or both. Therefore, GILZ levels may help identify at-risk populations for PTSD prior to additional traumatic exposures.

## Introduction

Post-traumatic stress disorder (PTSD) is a debilitating disorder that develops after exposure to a traumatic event^1–3^ in 7–12% of the population who eventually develop long-term symptomology, with a higher prevalence in females than males^4^. Individual symptom trajectories after exposure to trauma implicate both genetic and past environmental contributions, supporting the study of epigenetics in PTSD^5–7^.

Genes related to the hypothalamic–pituitary–adrenal (HPA) axis, glucocorticoid, and immune pathway are affected in response to a traumatic event^5,8–10^. Patients with severe PTSD show elevated levels of corticotropin-releasing factor (CRF). Changes in methylation on promoter regions of the glucocorticoid receptor (GR) are linked to PTSD symptoms and treatment efficacy^5,11^. Additionally, PTSD severity is dependent on the interaction between FK506 binding protein 51 (FKBP51), a GR co-chaperone, and early environmental factors such as childhood abuse^12–14^. Trans-generational PTSD shows FKBP51 epigenetic changes in both parent and offspring^15^ although the contribution of genetic versus environmental factors remains unclear. In addition, elevation of pro-inflammatory cytokines and high rates of comorbidity with autoimmune disorders and have also been observed^10,16^.

Glucocorticoid-induced leucine zipper (GILZ), encoded by the *Tsc22d3* gene and located on the X chromosome, is transcriptionally activated by glucocorticoids and has been associated with increased risk of PTSD symptoms^17^. GILZ has been extensively studied for its anti-inflammatory properties^18,19^, and now evidence is emerging on its potentially important role as a reliable indicator of glucocorticoid pathway sensitivity^19,20^. Post-mortem brain tissue studies of depressed patients show alterations in GILZ^21,22^ and rodent studies indicated that prolonged stress increases GILZ in the frontal cortex and hippocampus^23^.

We sought to determine the role of GILZ in the etiology of PTSD. To examine this, we assessed the genetic and epigenetic relevance of GILZ in blood samples from a clinical civilian cohort, the Grady Trauma Project (8, 20, 21). We assessed GILZ interactions with early-life stress, multiple stress exposures and current diagnosis. Our second objective was to compare changes in GILZ levels with other GR pathway genes in a mouse model of susceptibility to PTSD using a prenatal stressor with, or without, additional exposure to stress in adulthood^12–14^. Finally, additional manipulation of GILZ in vivo further validated its relevance to PTSD-like behaviors in mice after exposure to adulthood trauma.

We used two distinct stressors in the mouse model, (i) a prenatal stressor in late gestation, _CRF-induced_PNS^24^, a critical time window for stress reactivity with a vast effect on epigenetic programming^25,26^ and (ii) a mouse model for inducing PTSD in adulthood^27^, to understand the effect of epigenetic priming when the stress response is activated after early life development. In _CRF-induced_PNS we induced CRF release in the CSF of dams during late gestation^24,28^ causing stress-related behaviors by CRF modulation of the stress circuitry^29,30^ and initiation of the HPA axis^29^ without the added corticosterone elevation in response to handling.

The long-term effects of _CRF-induced_PNS on the brain and behavior of male and female offspring were assessed in adulthood, before or after exposure to the animal model of trauma. The following PTSD-related markers were then assessed: circulating corticosterone (CORT) levels and amygdalar expression levels of GR, FKBP51, CRF and GILZ.

## Materials/subjects and Methods

### Grady Trauma Project Cohort

#### Human samples

Participants in this study belonged to a larger study investigating the contribution of genetic and environmental factors in PTSD, the Grady Trauma Project. The institutional review boards of Emory University School of Medicine approved all study procedures and Grady Memorial Hospital and all subjects gave written informed consent to the study. The 135 male participants and 300 female participants were selected from a larger study, which has been described previously. Comparison groups were matched for gender, age, and ethnicity. There were no exclusion criteria^13,31^.

#### Clinical assessment

Briefly, the modified PTSD Symptom Scale (PSS), a psychometrically validated 17-item self-report scale assessing PTSD symptomatology over the prior 2 weeks, was filled out by participants.

The Clinician Administered PTSD Scale (CAPS) was also administered to a subset of 240 participants within 2–6 weeks after completing the screening assessment.

The traumatic events inventory (TEI) assesses lifetime history of trauma exposure and is our primary measure of both childhood abuse and non-childhood abuse trauma. For the measure of childhood abuse, two of the TEI questions assessed physical abuse and sexual abuse occurring before age 14 years. Based on these questions, 17.6% of the sample reported a history of childhood physical abuse and 18.8% reported a history of childhood sexual abuse. With these data, we created a three-level categorical variable reflecting number of types of childhood abuse: no childhood abuse (70.5% of sample), one type of either physical or sexual abuse (22.7%), or two types of both physical and sexual abuse (6.8%). (See Supplementary methods for more detail)

#### Microarray experiments and statistical analysis

The details for microarray experiments and statistical analysis of the gene expression and DNA methylation data for the Grady Trauma Project human samples are previously described^13^ (and in supplementary methods). In the current study, a candidate-based analysis for GILZ gene (four probes for gene expression and 15 CpGs for DNA methylation was performed. Briefly, raw microarray scan files from Illumina HT-12 v3.0 arrays (Illumina, San Diego, CA, USA) and Human Methylation 450k BeadChip (Illumina, San Diego, CA, USA) were exported using the Illumina Beadstudio program and loaded into R for downstream analysis (www.R-project.org). Evaluation of the different microarray steps was done using the Illumina internal controls. To correct for confounding as a result of batch effects, the data were normalized using an empirical Bayes method for batch correction. Reproducibility of the gene-expression and DNA methylation data was assessed using technical replicates, yielding average Pearson correlations of 0.99 for each. General linear models were constructed by regressing the gene-expression/DNA methylation betas against the PTSD group status and adjusting for sex, age, ethnicity, substance abuse, and treatment. The significance of association was estimated by two-tailed *P* values using the ANOVA *F* test. Results were corrected for multiple testing by 10,000 permutations using the permutation of regressor residuals test (http://cran.r-project.org/web/packages/glmperm/index.html).

### Experiment 1: _CRF-induced_PNS confirmation

#### Animals

Mice were maintained in a pathogen-free temperature-controlled (22 ± 1 °C) mouse facility on a reverse 12-h light–dark cycle (lights on at 18:00) at the Weizmann Institute of Science, according to institutional guidelines. Food and water were given ad libitum. All experimental protocols were approved by the Institutional Animal Care and Use Committee of The Weizmann Institute of Science.

#### _CRF-induced_PNS protocol

Eight-week-old ICR female mice (Harlan) were injected with a mixture of the two lentiviruses, the ‘Effector' and the ‘CRF-Target’, into the lateral ventricle^28^, as further described in Supplementary methods.

#### Breeding

Female ICR mice were mated at 11–15 weeks of age. Presence of a plug was checked before and after the dark cycle and then denoted day 0.5 of gestation. After breeding, the females were individually housed.

#### Experimental groups

Females with plug were randomly assigned to treatment groups to receive either doxycycline (Dox)-containing drinking water (0.5 mg/mL Dox and 0.2% sucrose) during late gestation, from 13.5-parturition, or to a control group receiving water without Dox during the same period. From day 18.5 of gestation, the females were checked twice a day for the presence of a litter (9:00–10:00, 17:00–18:00). Newborn litters found by 18:00 were designated as born on that day- postnatal day 0 (PND 0).

#### Offspring physiological measurements

*Litter size*: On PND 1, pups were counted and litters were culled to 10 pups (with sex distribution kept as equal as possible in each litter). Litters which had <7 pups were excluded. *Body weight*: For pre-weaning weights, total weights were averaged for the number of pups per litter. Post-weaning, pups were weighed individually at week 5, 6, and 10.

#### Maternal behavior

On days 6/7 and 17/18 postpartum, patterns of undisturbed nocturnal maternal behavior were observed during 160 min sessions. Each mother was observed every 15 min, for 1–3 s. This allowed the identification of the ongoing maternal behavior at the observation time. Various maternal and non-maternal behaviors were recorded in every observation. The score was “1” if the behavior occurred and “0” if it did not occur^24^. Maternal behavior measures included both self-(grooming, eating) and pup-directed behaviors (nursing, licking/grooming) and activity measures.

### Experiment 2: Susceptibility to PTSD-like behavior in _CRF-induced_PNS offspring PTSD induction protocol

After induction of the _CRF-induced_PNS in late gestation, mice were born and left undisturbed until 9–11 weeks of age. Then as previously demonstrated^27^ we induced PTSD in a subset of mice using stress enhanced fear learning (SEFL) adapted from Rau et al.^32^ and Rau and Fanselow^33^. On day one, in context A, mice receive 14 shocks of 1 mA with continuous pulse over 85 min in variable intervals, representing the pre-shocks. On day two, the same mice in context B receive five pulsed shocks of 0.7 mA over 5 min in fixed intervals. Shocks were given in a fear conditioning apparatus (TSE Systems, Bad Homburg, Germany).

Mice were then phenotyped in the following battery of tests: dark/light transfer to assess risk assessment, marble burying, startle and pre-pulse inhibition, and homecage locomotion (see supplementary methods). The SEFL exposure plus phenotyping is collectively called ‘PTSD induction’.

From the behavioral phenotyping, subpopulations of PTSD-like were extracted from the SEFL exposed group based on extreme behavior or lack thereof. Mice were given a score for each test based on inclusion into lowest 20% of risk assessment time, latency to peak startle amplitude (reaction time to the startle stimulus), percent pre-pulse inhibition response reduction, and upper 20% of activity in non-active phase of the dark/light cycle and percent of marbles buried. Mice that were in the upper or lower 20% boundary, depending on the test, in at least three out of five behavioral tests, were categorized as “PTSD-like”, as described in Lebow et al.^27^ and further discussed in supplementary methods.

#### Corticosterone collection and measurement

Plasma was extracted from blood samples of male mice three weeks after the trauma protocol. Samples were collected by tail bleed under basal conditions, 30 min after insertion into restraint tubes. All blood samples were collected at 13:00, 8 h after the beginning of the dark phase. Blood samples were centrifuged immediately (3500 rpm for 25 min at 4 °C) and extracted plasma was stored at −80 °C until assayed for corticosterone using a radioimmunoassay corticosterone kit (MP biomedicals, Solon, OH, USA)

#### Brain tissue collection

Immediately after decapitation, the brain was removed and placed in a steel brain matrix, 1.0 mm, coronal (model: 51386: Stoelting Co. Wood Dale, IL, USA). The brains were sliced into 2 mm slices using standard razor blades and were quickly frozen on dry ice. The area of interest was punched out using a microdissecting needle of 16 G for the amygdala. Punches were immediately stored at −80 °C.

#### RNA extraction and real-time PCR

Samples for RNA extraction were collected from PTSD-like controls (*n* = 5) and _CRF-induced_PNS PTSD-like mice (*n* = 7) during brain microdissection with inclusion criteria threshold over 4. RNA extraction was performed using a 5 PRIME PerfectPure RNA Cell & Tissue kit (5 Prime GmbH, Hilden, Germany) RNA preparations were reverse transcribed to generate cDNA using High Capacity cDNA Reverse Transcription Kit (Applied Biosystems, Waltham, MA, USA). The cDNA products were used as templates for Real-Time PCR analysis.

CRF 5′ gcagttagctcagcaagctcac, caaatgatatcggagctgcg

GR 5′ TGCTGTTTATCTCCACTGAATTACA, TCCTTAGGAACTGAGGAGAGAAGC

FKBP51 5′ATGACTACTGATGAGGGCAC, GACATAAACTTTGTCACCAAAC

GILZ 5′ CAGCCTACTCCTTGCTTCAGGGC, TTCATGGTTCGGTTGCCGGGG

GILZ primers target mouse variants 1 and 2, as described^34^. Real-time PCR reactions were carried out on a 7500 Real-time PCR system using fluorescent SYBR Green technology (Applied Biosystems, Waltham, MA, USA) for full protocol see supplementary methods.

#### Bioinformatics

Prediction of GRE regions of GILZ as targets for pyrosequencing was done using the UCSC genome browser^35^ to define the genomic region and the MatInspector program^36^, from the Genomatix Genome Analyzer package (Genomatix Software GmbH, Germany) for prediction of potential transcription factor binding sites.

#### DNA extraction and pyrosequencing

Samples for DNA extraction were collected from PTSD-like controls (*n* = 5) and late gestation PTSD-like mice (*n* = 7) during brain microdissection. DNA extraction was then performed (see supplementary methods) and the DNA was then sent to the Barts and the London Genome Centre, London, UK for pyrosequencing.

### Experiment 3: Anxiety-like behavior in adult _CRF-induced_PNS offspring without PTSD-induction (without SEFL)

Mice underwent the _CRF-induced_PNS protocol and then the offspring were left undisturbed until week 9–11. At that point they were phenotyped behaviorally in the following battery of tests: open field, light/dark transfer test, and elevated plus maze (see supplementary method).

### Experiment 4: Anxiety-like behavior in _CRF-induced_PNS offspring with no PTSD-induction (SEFL) in females and Susceptibility to PTSD-like behavior in _CRF-induced_PNS offspring in females

Protocols as described in experiments 2 and 3.

### Experiment 5: Knockdown of GILZ by lentiviral infection in the basolateral amygdala in adulthood

#### Endogenous GILZ staining

Animals were anaesthetized and then perfused with phosphate-buffered 4% paraformaldehyde. Staining for endogenous GILZ was performed using α-GILZ/TILZ (Abcam, ab55015), 1:100.

#### shGILZ lentivirus production and confirmation

Four knockdown psiHIV-H1 vectors containing GILZ (Tsc22d3) shRNA target sequences or a scrambled control were obtained from Genecopoeia (Rockville, MD, USA). Vectors were amplified and transfected. Infectious lentiviruses were harvested at 48 and 72 h following transfection, filtered through 0.45 μm-pore cellulose acetate filters, concentrated by ultracentrifugation, re-dissolved in sterile HBSS, aliquoted and stored at −80 °C. Vector concentrations were analyzed using eGFP fluorescence in HEK293T cells infected with serial dilutions of the recombinant lentivirus. AR-5 cells (as treated in Lalmansingh et al.^37^) were then infected with 3 different viruses at varying concentrations and control virus. RNA was extracted after 5 days (QIAGEN RNA extraction kit, QIAGEN, Hilden, Germany). Real time PCR was performed on cDNA and normalized to both HPRT and GFP in order to determine the overall downregulation and proportion of cells infected.

#### Intracerebral injections of shGILZ lentiviral vectors

Adult (8 weeks) ICR male mice (Jackson ImmunoResearch Laboratories, West Grove, PA, USA) received bilateral stereotaxic injections of lentivirus carrying either shGILZ or control shRNA to the BLA. Mice were anesthetized using 1.5% isofluorane and were bilaterally injected with 1 µl lentiviral preparation into BLA on each side. Lentiviral injections were conducted using a computer-guided stereotaxic instrument and a motorized nanoinjector (Angle Two^TM^ Stereotaxic Instrument, myNeurolab, St. Louis, MO, USA), which is fully integrated with the mouse brain Paxinos atlas via a control panel. Bregma coordinates were as follows: AP ± 1.34, ML ± 3.3, DV-4.8. Mice were given a 2 week period for recovery before behavioral testing.

#### Immunohistochemistry and histological confirmation of all injected brains

Fixed brains perfused in dams on day E17.5, or in adult males after GILZ knockdown (KD) injection and phenotyping were serially sectioned and confirmation of the accuracy of the injection site was done by immunostaining using biotinylated α-GFP antibody raised in goat as primary antibody (Abcam ab6658, Cambridge, UK) and streptavidin conjugated Cy2 anti rabbit as secondary antibody (Jackson Immunoresearch laboratories Inc, West Grove, PA, USA).

#### Quantification of degree of infection

Slices stained for α-GFP were chosen for each mouse in the GILZ KD injected groups. Degree of infection was assessed on a scale of 1–5 for each injected side and then totaled based on the breadth of infection. Unilateral BLA was determined to be about 100–120 mm^2^ on average. Scoring: 1 = minimal infection, i.e., <5 mm^2^, 2 = <10 mm^2^, 3 = <25 mm^2^, 4 = <50 mm^2^, 5 = >50 mm^2^. One animal was excluded due to GFP staining in the hippocampus and septum without BLA GFP staining.

#### Statistical analysis

Data were expressed as mean ± standard error of the mean (SEM). Data were analyzed using SPSS (SPSS Inc., Chicago, IL, USA). All data sets were tested for departures from normality with Shapiro–Wilks test. Students *t*-test or Mann–Whitney was used for all comparisons between two groups. Levine test was used to assess equal variance in statistical analyses between two groups. ANOVA or Kruskal-Wallis H test were used for comparing multiple groups. All data sets were corrected for multiple comparisons and a two-factor univariate ANOVA was used when necessary. Three-way ANOVA was conducted on all amygdala mRNA data, as described. Kruskal-Wallis was used when samples were non-parametric. Dunn’s pairwise comparisons, student *t*-test and Bonferroni comparisons were used as post-hocs. Linear regression and r values were determined in SPSS. *P < 0.05, **P < 0.01 and ***P < 0.001.

## Results

### Changes in human GILZ mRNA gene expression in the blood reflect PTSD status, severity of trauma, and in males only also the number of traumatic exposures

**Fig. 1:**
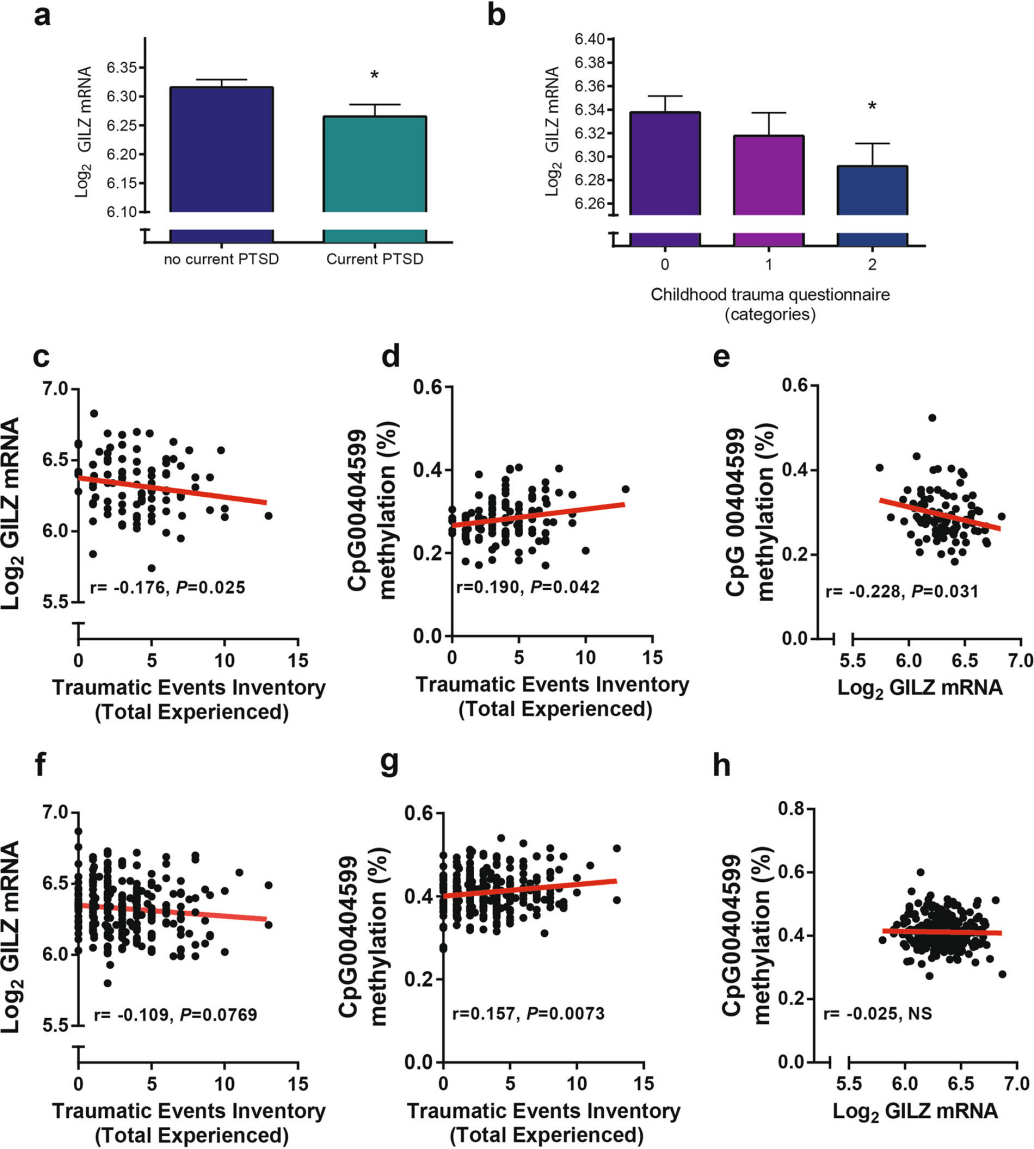
GILZ mRNA level is modified in blood of human PTSD patients in response to PTSD status, severity and quantity of trauma and is epigenetically regulated. **a** Gene expression array shows significantly reduced GILZ mRNA with current PTSD (*P* *=* 0.047) (*n* = 396). **b** An increasing number of types of moderate to severe childhood abuse (emotional, sexual and/or physical) is associated with reduced levels of GILZ mRNA (*P* *=* 0.0491). **c** Male GILZ mRNA levels are negatively correlated with Traumatic Events Inventory score (*P* = 0.025, *n* = 105). **d** CpG methylation (Beta values) in the 5′UTR of GILZ and total events inventory show a positive correlation (*P* *=* 0.042, *n* = 123) in males and **e** the same CpG methylation (Beta values) methylation and mRNA levels are inversely correlated in males only (*P* = 0.031, *n* = 276). **f** Female GILZ mRNA levels have a tendency toward significance with Traumatic Events Inventory score (*P* *=* 0.077, *n* = 316). **g** CpG methylation (Beta values) in the 5′UTR of GILZ and total events inventory show a positive correlation (*P* *=* 0.0073, *n* = 316) in females. **h** CpG methylation (Beta values) and mRNA levels do not correlate in females (*n* = 276)

First, we assessed changes in GILZ mRNA in blood samples of male and female PTSD patients using human gene expression array data. Of four probes for GILZ mRNA, the probe closest to the 5’UTR showed significantly reduced expression in association with current PTSD (*P* = 0.047, Fig. 1a). Exposure to different types of moderate to severe childhood abuse (physical, sexual, and/or emotional abuse) was also significantly associated with reduced levels of GILZ mRNA (*P* = 0.0491, Fig. 1b). A correlation was also demonstrated between CAP score and reduced GILZ mRNA levels (*P* = 0.0160), Supplementary Fig. 1a). Methylation on the N-shore of the promoter CpG island was found to be also positively correlated with CAP score (*P* = 0.00941, Supplementary Fig. 1b). Percentage Methylation on the same CpG was found to be correlated with the PSS score (*P* = 0.0488, Supplementary Fig. 1c).

The data was then split by gender to assess the impact of the gene location on the X chromosome. No differences were found based on gender in either the CAPS score or PSS scores. However, differences in methylation and GILZ mRNA levels with the lifetime total number of traumatic events experienced in adulthood or the traumatic events index (TEI) were observed.

In males only, GILZ mRNA levels were significantly negatively correlated with the lifetime total number of traumatic events experienced in adulthood (*P* = 0.025, Fig. 1c). Additionally, methylation of an intronic CpG between the first and second exons of the gene showed a significant positive correlation with total traumatic events experienced (*P* = 0.042, Fig. 1d). Next, we found that GILZ mRNA levels were significantly inversely correlated with the % methylation (*P* = 0.031, Fig. 1e), suggesting regulation by methylation. In contrast, the number of traumatic events was not correlated with mRNA levels, though there was a positive correlation in women with % methylation of the same intronic site. As a result, there was no significant correlation between mRNA levels (Fig. 1f) and % CpG methylation (Fig. 1g), suggesting that the regulation by methylation occurs in males only (Fig. 1h). Therefore, while GILZ mRNA levels are significantly lower in male and female PTSD patients and in those with severe childhood trauma and increased symptomology, lifetime TEI correlation with GILZ mRNA was only significant and epigenetically regulated in males.

In order to further understand the contribution of GILZ to PTSD, we tried to model the effect of the correlation found in males by studying offspring of both sexes. Using multiple established mouse models, we sought to specifically understand the mechanism by which multiple lifetime traumas affect GILZ expression and its epigenetic regulation or by which one early-life trauma predisposes to additional trauma later in life.

### Experiment 1: Confirmation of _CRF-induced_PNS shows shorter length of gestation, but does not alter postnatal pup weight nor postnatal maternal behavior

We chose an very early-life stressor in late gestation in accordance with the Barker’s hypothesis, which states that the uterine environment influences the development of disease in adulthood^38^. This hypothesis has been confirmed in both clinical studies and animal models with respect to psychiatric disorders^39–41^.

**Fig. 2:**
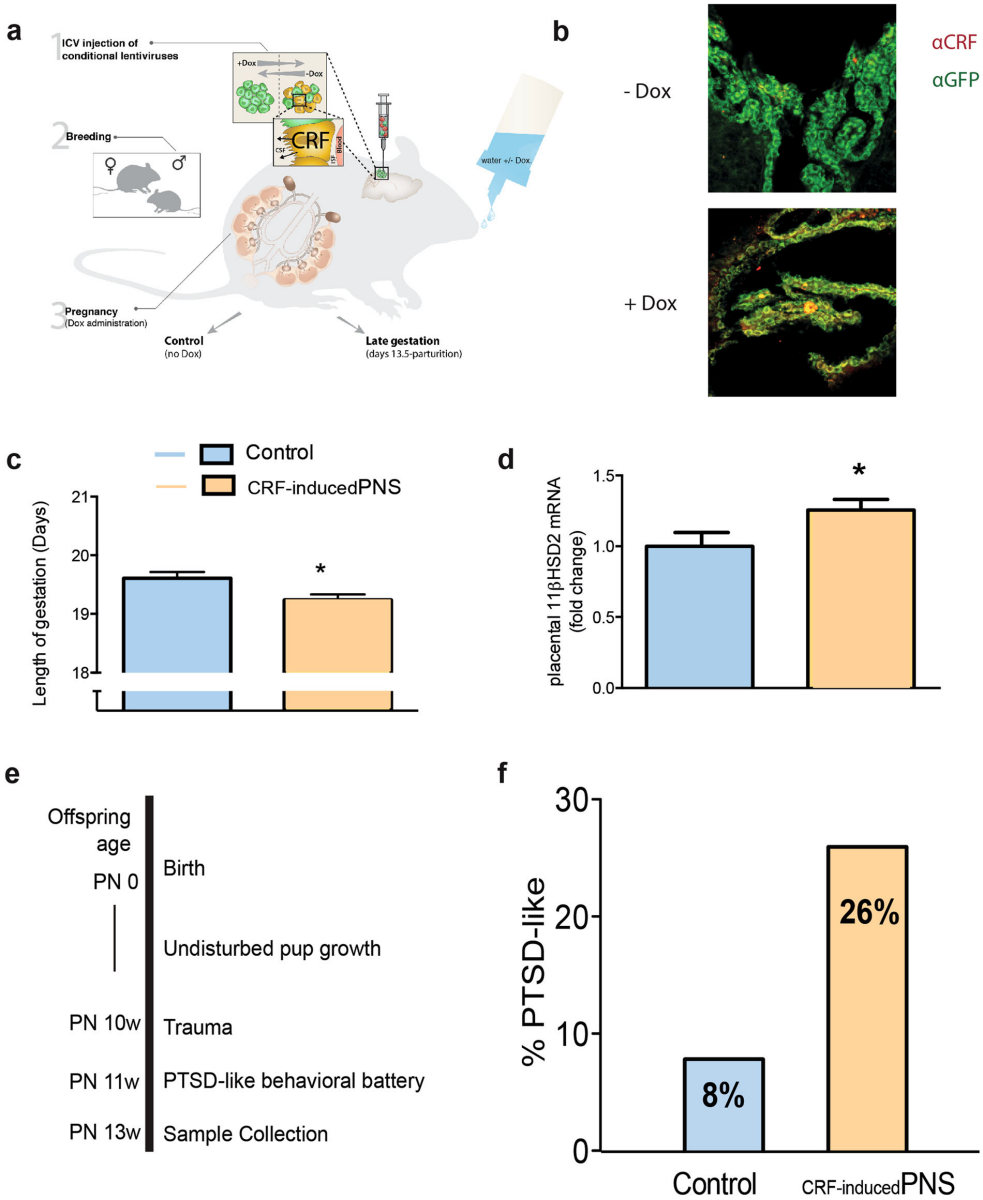
_CRF-__induced_PNS affects length of gestation, placental protection against corticosterone and increases pups’ susceptibility to PTSD-like behavior in adulthood. **a** Schematic representation of the animal model used for _CRF-__induced_PNS. Female virgin mice were ICV injected with conditional viruses (1) and following recovery were bred (2). Doxycycline (Dox) was given in their drinking water in late gestation (3). **b** Representative immunostaining GFP and CRF of maternal brain removed at E17.5 of pregnancy without Dox, top and with Dox, bottom. **c** Pregnant dams exposed to _CRF-induced_PNS (*n* = 5) gave birth significantly earlier than controls (*n* = 7, *P* = 0.015]. **d** Placental 11βHSD2 mRNA levels are significantly higher in _CRF-induced_PNS mice versus controls. Independent samples *t*-test indicated that _CRF-induced_PNS significantly increased 11bHSD levels in the dams (*P* *=* 0.047). **e** Timeline of post-natal (PN) experiments. After weaning, pups were left undisturbed until adulthood, then underwent an exposure to “Trauma” followed by behavioral anxiety test, corticosterone collection and brain microdissection. Behavioral data from the PTSD-like battery divided the mice into ‘Resilient' versus ‘PTSD-like' with a background of CRF-exposure or not. Two-way ANOVA with multiple comparisons was used. **f** In all, 81 male pups underwent “Trauma” in adulthood. Comparing the distribution of mice categorized as displaying a PTSD-like phenotype across the Control and _CRF-induced_PNS groups (supplementary table 1) indicated that _CRF-induced_PNS increased the susceptibility to PTSD-like behavior in adulthood from 8 to 26%, (three out of 35 mice and 12 out of 46 mice, respectively) [*Χ*^2^ Continuity Correction _(1)_ = 2.964; *P* = 0.042; *φ* = 0.223; *P* *=* 0.044]. *P *<* 0.05

As such we utilized the previously established _CRF-induced_PNS^24^ model based on the established model of choroid plexus induction of CRF^28^. Specifically, female virgin mice were injected intracerebroventricularly (Fig. 2a) with a combination of two lentiviral vectors^24^. Following a two-week recovery period, these females were mated and then the pregnant dams were administered Dox, or not, on day E13.5 until end of gestation (Fig. 2a). The Dox administration throughout the third trimester activated the lentiviral vectors to produce continuous overexpression of CRF by the cells in choroid plexus and to its distribution throughout the CNS by the CSF circulation.

In a separate cohort of dams, we confirmed the induction of CRF in the choroid plexus cells, immunohistochemistry staining for CRF (red) and GFP (viral infection marker, green) in maternal brains with or without Dox induction was performed on brains removed on day E17.5, 4 days after Dox administration (Fig. 2b). Similar to previously reported^28^, Dox-treated dams showed robust CRF immunoreactivity in the choroid plexus cells.

_CRF-induced_PNS dams gave birth earlier than control dams (*P* < 0.05, Fig. 2c). However, there were no differences in maternal behavior in the 1 week post-partum (Supplementary Fig. 2a) nor in the 3 week postpartum (Supplementary Fig. 2b). The _CRF-induced_PNS pups showed no differences in weight pre- or post-weaning (Supplementary Fig. 2c). Interestingly, placental 11βHSD2 mRNA, an enzyme that converts CORT to its inactive form, was mildly upregulated on E17.5 (Fig. 2d, *P* = 0.047) in males only (female data was not significant). Previous evidence from this model showed maternal increases in corticosterone after Dox administration in late gestation^24^, demonstrating early dysregulation of glucocorticoid system. 11βHSD2 mRNA levels demonstrate an attempt by the mother to protect the fetuses^42^.

### Experiment 2: _CRF-induced_PNS in late gestation increases the likelihood to exhibit PTSD-like behaviors following exposure to adulthood trauma in male mice

Following weaning, male mice and female were separated and left undisturbed until adulthood. Between 9 and11 weeks, 81 male adult offspring were exposed to the PTSD induction protocol and then tested in a behavioral battery to determine a PTSD-like subpopulation of mice (Fig. 2e). The number of PTSD-like mice per treatment group is presented as percentages (Fig. 2f). Subcategorization of mice was determined by extreme behavior in risk assessment in dark-light transfer test, percentage marble burying, latency to peak startle amplitude, percentage pre-pulse inhibition (ppi), and home-cage locomotion(Supplementary Table 1)^27^. The _CRF-induced_PNS mice were three times more likely to develop PTSD-like symptoms (26%) than the unexposed, control mice (8%) (*P* < 0.05). _CRF-induced_PNS increases the number of male mice displaying PTSD-like symptoms after adulthood trauma, reaffirming late gestation as an important sensitive period in fetal development that affects response to stressful challenges later in life.

### Experiment 3: Behavior of _CRF-induced_PNS offspring without exposure to “PTSD induction” in adulthood in male mice

In order to better elucidate the underlying mechanism of increased susceptibility to PTSD-like behaviors, we sought to disassociate the effects of _CRF-induced_PNS and exposure to PTSD induction in adulthood, on anxiety-like behaviors. Four groups were included: (1) Control + no PTSD induction, (2) _CRF-induced_PNS + no PTSD induction, (3) Control + PTSD-like (after exposed PTSD induction), and (4) _CRF-induced_PNS + PTSD-like (after exposure to PTSD induction, Supplementary Figure 3a). In the first two groups, “basal” anxiety was assessed in a battery of tests and indicated no significant differences in anxiety-like behaviors between control + no PTSD induction and _CRF-induced_PNS + no PTSD induction mice, as assessed in the open field (Supplementary Fig. 3b), dark-light transfer (Supplementary Fig. 3c) and elevated plus maze (Supplementary Fig. 3d) tests. These findings suggest that _CRF-induced_PNS does not affect “basal” anxiety levels.

### Experiment 4: _CRF-induced_PNS does not affect anxiety-like behaviors or susceptibility to PTSD in adulthood among female mice

Two experiments were carried out in adult female offspring. The first assessed the effects of _CRF-induced_PNS on anxiety-like behaviors in adulthood and indicated that in a similar manner to that found in adult male mice, _CRF-induced_PNS alone, without exposure to any additional stressors, did not affect anxiety-like indices in the open field, dark–light transfer and elevated plus maze tests (Supplementary Fig. 4a–c).

The second experiment assessed the effects of _CRF-induced_PNS on the consequences of undergoing the PTSD induction procedure, as assessed by the anxiety-like behaviors battery (Supplementary Fig. 5a–e). In contrast to males, _CRF-induced_PNS did not affect susceptibility to PTSD in adult female mice; _CRF-induced_PNS exposed and Control adult female mice exhibited similarly high rates of mice displaying PTSD-like behaviors (Supplementary Fig. 5f). Since females in general have a higher prevalence of PTSD in humans^43^, and show greater anxiety-like phenotypes in mouse models of PTSD^44^, it is suggested that the PTSD induction protocol may have created a ceiling effect.

### The increased susceptibility to develop PTSD among _CRF-induced_PNS exposed adult male mice is associated with GR pathway-related markers, most specifically with amygdalar GILZ mRNA levels: (Experiments 2 and 3)

**Fig. 3:**
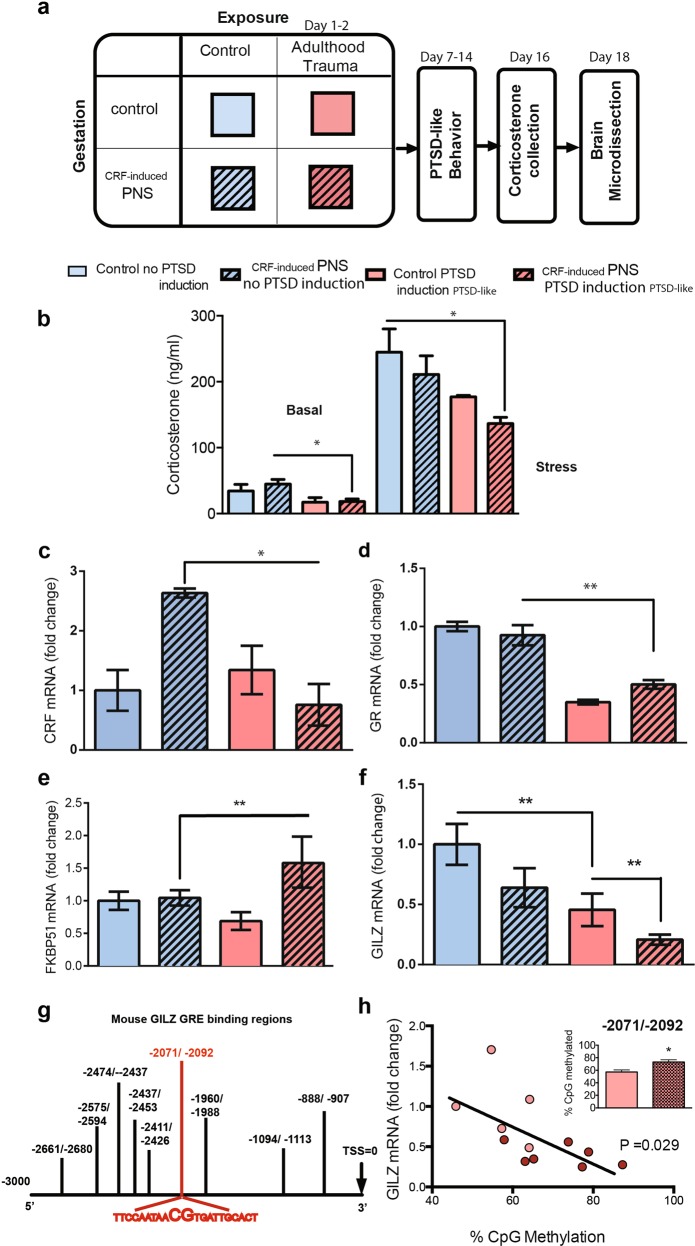
Amygdalar GILZ levels are downregulated following _CRF-induced_PNS and PTSD induction exposure; GILZ is epigenetically regulated. **a** Schematic representation of experimental groups and timeline. Groups in blue (no adult trauma) underwent anxiety behavioral battery and orange groups (with adult trauma) underwent PTSD behavioral battery testing after exposure to trauma and were categorized as PTSD-like. **b** Basal (left panel): A Kruskal-Wallis H test indicated that there was a statistically significant difference in basal CORT levels between the different groups [*Χ*^2^_(3)_ = 11.736; *P* = 0.008], with a mean rank basal CORT level of 11.20 for Control Trauma, 12.33 for _CRF-induced_PNS Trauma, 20.06 for Control no Trauma and 25.61 for _CRF-induced_PNS no Trauma. Dunn’s corrected pair-wise comparisons indicated that the _CRF-induced_PNS Trauma group basal CORT levels were significantly lower than those of the _CRF-induced_PNS no Trauma (*P* = 0.015); all other pair-wise comparisons were not significant. Stress: A Kruskal-Wallis H test indicated that there was a difference in stress-induced CORT levels between the different groups [*Χ*^2^_(3)_ = 8.693; *P* = 0.034], with a mean rank of 8.60 for _CRF-induced_PNS Trauma, 14.50 for Control Trauma, 17.17 for _CRF-induced_PNS no Trauma and 19.50 for Control no Trauma. Dunn’s corrected pair-wise comparisons indicated that the _CRF-induced_PNS Trauma group Stress CORT levels were lower than those of the Control no Trauma (*P* = 0.043); all other pair-wise comparisons were not significant. **c** CRF mRNA, *n* = 6–8 levels were significantly affected only by “PTSD induction” [*F*_(1,33)_ = 4.706; *P* *=* 0.037]; the interaction “_CRF-induced_PNS” × “PTSD induction” was significant [*F*_(1,33)_ = 4.311; *P* = 0.046]. Subsequent *t*-test comparisons [Bonferroni corrected for multiple comparisons significance set at 0.05/4 = 0.0125] indicated that amygdalar CRF mRNA levels were affected by “PTSD induction” when preceded by CRF-exposure [_CRF-induced_PNS Trauma < _CRF-induced_PNS no Trauma: *t*_(20)_ = −3.078; *P* = 0.006]. **d** GR levels were significantly affected only by “PTSD induction” [*F*_(1,35)_ = 12.303; *P* = 0.001]; Subsequent t-test comparisons [Bonferroni corrected for multiple comparisons significance set at 0.05/4 = 0.0125] indicated that amygdalar GR mRNA levels, *n* = 6–8, were affected by “PTSD induction” when preceded by CRF-exposure [_CRF-induced_PNS Trauma < _CRF-induced_PNS no Trauma: *t*_(20)_ = 3.528; *P* *=* 0.002]. **e** Amygdalar FKBP51 mRNA levels, *n* = 6–8 were significantly affected only by “PTSD induction” [*F*_(1,33)_ = 4.434; *P* = 0.043]; Subsequent *t*-test comparisons [Bonferroni corrected for multiple comparisons significance set at 0.05/4 = 0.0125] indicated that amygdala FKBP51 levels were only affected by “PTSD induction” if preceded by _CRF-induced_PNS [_CRF-induced_PNS Trauma > _CRF-induced_PNS no Trauma: *t*_(18)_ = 3.540; *P* = 0.002]. **f** Interestingly, amygdalar GILZ mRNA levels (*n* = 6–8) were significantly affected by both “_CRF-induced_PNS” [*F*_(1,35)_ = 15.571; *P* = 0.000] and “PTSD induction” [*F*_(1,35)_ = 17.158; *P* = 0.000]; the interaction “_CRF-induced_PNS” × “PTSD induction” was not significant [*F*_(1,35)_ = 1.907; *P* = 0.176]. Subsequent *t*-test comparisons [Bonferroni corrected for multiple comparisons significance set at 0.05/4 = 0.0125] indicated that amygdala GILZ levels were significantly affected by “PTSD induction” in both control mice [Control Trauma < Control no Trauma: *t*_(16)_ = 3.601, *P* = 0.002], and _CRF-induced_PNS [_CRF-induced_PNS Trauma < Control Trauma: *t*_(24)_ = −4.185; *P* = 0.000]. **g** There were nine putative GRE sites identified by bioinformatics prediction. However, only one GRE site which contains a CG, -2071/-2092. **h** GILZ mRNA levels are negatively correlated with %CpG methylation (*r* = −0.625; *P* = 0.029). Light circles represent the control Trauma group and dark circles represent _CRF-induced_PNS Trauma group. *P < 0.05, **P < 0.01. Data shown are mean ± SEM

After behavioral phenotyping (Fig. 3a), mice from each of the four groups underwent assessment of plasma CORT levels, a marker of HPA axis changes in human PTSD^45^, both basally and in response to 25 min of restraint stress as described in^27^. The _CRF-induced_PNS + PTSD-like group had significantly lower basal CORT levels (Fig. 3b left, *P* = 0.015) than the _CRF-induced_PNS + no PTSD induction. After restraint, CORT levels were also significantly lower in _CRF-induced_PNS + PTSD-like mice as compared with Control + no PTSD induction (Fig. 3b right, *P* = 0.043).

Assessing amygdalar GR, CRF, FKPB51 and GILZ mRNA with or without _CRF-induced_PNS and with or without PTSD induction, allowed assessment of the impact of the stressors independently or in combination, on amygdalar PTSD-related GR transcripts.

CRF mRNA levels were significantly affected only by PTSD induction (*P* *=* 0.037); the interaction _CRF-induced_PNS × PTSD induction was significant (*P* = 0.046). Student’s *t*-tests, Bonferroni corrected for multiple comparisons, indicated that amygdalar CRF mRNA levels were affected by PTSD induction when preceded by CRF-exposure (*P* = 0.006, Fig. 3c).

GR levels were significantly affected only by PTSD induction (*P* = 0.001); *T*-test comparisons Bonferroni corrected indicated that amygdalar GR mRNA levels were affected by PTSD induction when preceded _CRF-induced_PNS (*P* *=* 0.002, Fig. 3d).

Amygdalar FKBP51 mRNA levels (*n* = 6–8) were significantly affected only by PTSD induction (*P* = 0.043); *t*-test comparisons, Bonferroni corrected, indicated that amygdala FKBP51 levels were only affected by PTSD induction if preceded by _CRF-induced_PNS *(P* = 0.002, Fig. 3e).

Interestingly, amygdalar GILZ mRNA levels (*n* = 6–8) were significantly affected by both _CRF-induced_PNS (*P* = 0.000) and PTSD induction (*P* = 0.000); the interaction _CRF-induced_PNS × PTSD induction was not significant (*P* = 0.176). *T*-test comparisons, Bonferroni corrected, indicated that amygdala GILZ levels were significantly affected by PTSD induction in control mice (*P* = 0.002) and _CRF-induced_PNS (*P* = 0.000, Fig. 3f). In mice exposed to the combination of the two stressors, GILZ mRNA levels were lowest.

In order to elucidate the impact of each of the two stressors, _CRF-induced_PNS and PTSD induction, independently or in combination, on amygdalar GILZ, FKBP51, GR, and CRF, three-way ANOVA analyses were performed between the following factors: “_CRF-induced_PNS” (Control/ _CRF-induced_PNS), “PTSD-induction” (No PTSD induction/‘Adulthood PTSD induction’); With-in factor with repeated measures: ‘amygdala levels’ (GILZ/FKBP51/GR/CRF). In addition, all the interactions were assessed: “_CRF-induced_PNS” × “amygdala levels”, “PTSD induction” × “amygdala levels”, “_CRF-induced_PNS” × “PTSD induction” and “_CRF-induced_PNS” × “PTSD induction” × “amygdala levels”.

Table 1 summarizes these two-factor analyses and outlines the distinct patterns of effects across the GR pathway-related markers. In summary, CRF, GR and FKBP51 mRNA levels were significantly reduced following PTSD induction only among _CRF-induced_PNS mice. However, amygdala GILZ mRNA levels were significantly affected by both _CRF-induced_PNS and PTSD induction. Out of all the amygdalar GR pathway-related markers tested, GILZ mRNA had the highest percent of variance explained in both types of exposures, _CRF-induced_PNS (30.8%) and adulthood PTSD induction (32.9%).

**Table 1:** GILZ has the highest variance explained among all amygdalar PTSD-related genes

		_CRF-induced_PNS	PTSD induction PTSD-like	Interaction
GILZ	Sig difference	Cntrl > _CRF-induced_PNS, *P* = 0.000**	NoPTSDI > PTSDI, *P* = 0.000**	NS, *P* = 0.176
	% Variance explained	**30.8%**	**32.9%**	5.2%
FKBP51	Sig difference	Cntrl < _CRF-induced_PNS, *P* = 0.087~	NoPTSDI < PTSDI, *P* = 0.043*	NS, *P* = 0.147
	% Variance explained	8.6%	11.8%	5.3%
GR	Sig difference	NS, *P* = 0.364	NoPTSDI > PTSDI, *P* = 0.001**	*P* = 0.055~
	% Variance explained	2.4%	**26.0%**	10.1%
CRF	Sig difference	NS, *P* = 0.342	NoPTSDI > PTSDI *P* = 0.037 *	*P* = 0.045*
	% Variance explained	2.1%	12.5%	11.6%

Separate Two-way ANOVA [“_CRF-induced_PNS” (Control/_CRF-induced_PNS) and “PTSD induction” (No PTSD induction/ PTSD induction) and their interaction “_CRF-induced_PNS” × PTSD induction] were conducted for each of the PTSD-related genes. These two-way analyses, indicated that CRF, GR, and FKBP51 levels were significantly altered following PTSD induction only among _CRF-induced_PNS mice; downregulating both CRF (*P* = 0.006) and GR (*P* = 0.002), while upregulating FKBP51 (*P* = 0.002). However, amygdalar GILZ mRNA levels were significantly down regulated by both CRF exposure (_CRF-induced_PNS PTSD induction < _CRF-induced_PNS no PTSD induction; *P* = 0.000) and PTSD induction (Control PTSD induction < Control no PTSD induction; *P* = 0.008). Moreover, the percentage of variance explained in amygdalar GILZ levels were more than 30% for the effects of both “CRF exposure” (_CRF-induced_PNS) and “PTSD induction” in adulthood, while the variance explained in amygdalar CRF, FKBP51 and GR was restricted to “PTSD induction” in adulthood alone and ranged between 12 and 26%

*Cntrl* control, *noPTSDI* no PTSD induction (in adulthood), *PTSDI* PTSD induction (in adulthood), *NS* non-significant

### GILZ is epigenetically regulated

To better understand the mechanism of GILZ regulation following two stress exposures, _CRF-induced_PNS and/or PTSD-induction in adulthood, we examined epigenetic changes in the promoter region of the GILZ gene (*Tsc22d3*). A total of nine putative GRE sites were predicted using the MatInspector program. However, only one site contained a CG region. Interestingly, this specific methylation site on the GRE site -2071/-2092 is part of a cluster of GRE sequences in the GILZ promoter region^46^ containing highly conserved human GRE regions^47^ (Fig. 3g). A significant increase was observed in the methylation levels between _CRF-induced_PNS + PTSD induction (mice exposed to two traumas: one in late gestation and one in adulthood) as compared with Control + PTSD induction (mice exposed to trauma only in adulthood; Figure 3h, inset). Furthermore, a significant correlation (*r* = −0.625; *P* = 0.029) was observed between GILZ GRE methylation (% CpG methylation) and GILZ mRNA expression levels (Fig. 3h). This data further demonstrates that stress associated reduction of GILZ is regulated by methylation as seen in Grady Trauma Project Cohort.

### Amygdalar GILZ mRNA in males and females after _CRF-induced_PNS only: comparison of experiments 3 and 4

After we identified GILZ as a possible predictive factor in males, we returned to the males who underwent _CRF-induced_PNS only and tried to ascertain if there was already a predisposition in males only to alterations in GILZ. Males levels of amygdalar GILZ mRNA were lower than females levels (Supplementary Fig. 6). There was a significant main effect of sex on GILZ mRNA levels with _CRF-induced_PNS alone (*P* = 0.000). In addition, effect of _CRF-induced_PNS had a very strong tendency toward significance (*P* = 0.051), meaning that _CRF-induced_PNS, one stressor, had more of an impact on males than females.

### Experiment 5: Lentiviral-mediated knockdown of GILZ mimics PTSD-like behavior in adult mice

**Fig. 4:**
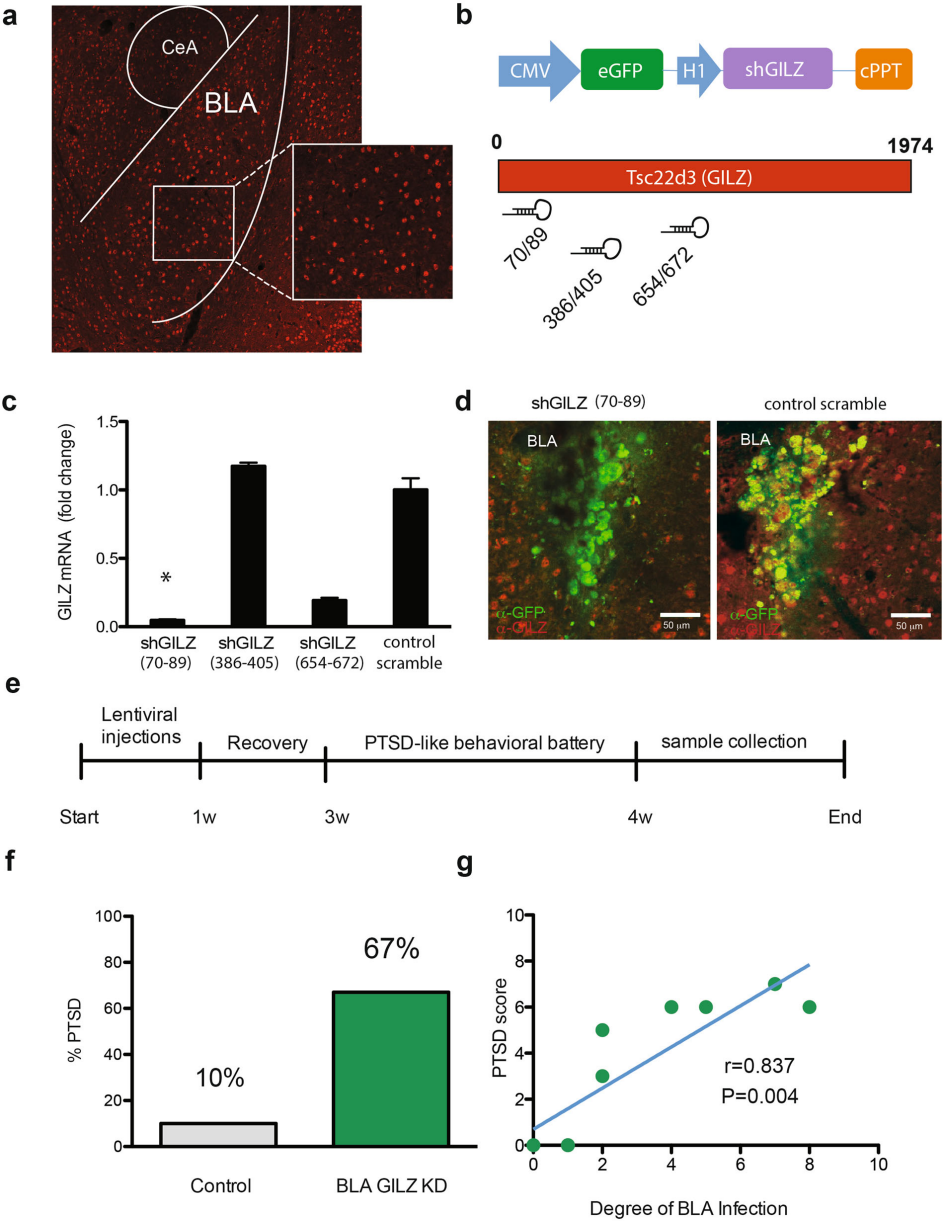
BLA GILZ knockdown increases susceptibility to PTSD-like behavior. **a** GILZ immunoreactivity in the basolateral amygdala (BLA) and central amygdala (CeA). **b** Schematic representations of the lentiviral construct designed to knockdown (KD) mouse GILZ and express eGFP reporter (upper panel) and the three different shRNA target sequences designed from the open reading frame of the mouse *Tsc22d3* (GILZ) gene. **c** Three different shRNA target sequences from the open reading frame of the mouse GILZ gene, driven by the H1 promoter and carrying a GFP reporter were compared for GILZ KD efficiency along with a scrambled control, in amygdala AR-5 cells. A Kruskal-Wallis H test indicated a statistically significant difference in GILZ mRNA levels between the different viruses [*Χ*^2^_(3)_ = 11.00; *P* = 0.012] when normalized to GFP mRNA. Dunn’s corrected pair-wise comparisons indicated that the GILZ mRNA levels of the GILZ 70–89 virus were lower than those of the control virus (CV) (*P* = 0.041), the GILZ 654–672 virus (*P* = 0.041) and GILZ 386–405 virus (*P* = 0.041); all other pair-wise comparisons were not significant. Data shown are mean ± SEM. **d** In vivo illustration of shGILZ (70–89) lentiviruses inducing GILZ silencing. Brain slices of mouse injected with shGILZ (70–89) (left panel) or control (right panel) viruses were double immunostained for GILZ and GFP. Red-stained cells represent endogenous GILZ and green stained cells represent lentiviral GFP immunoreactivity. **e** Experimental timeline of GILZ KD injections and PTSD-susceptibility tests. **f** 67% of BLA-GILZ KD injected mice were categorized as PTSD-like as opposed to 10% of CV [Χ^2^ Continuity Correction _(1)_ = 4.328; *P* = 0.018; *φ* = 0.587; *P* = 0.011]. **g** In addition, a strong (r = 0.837) and significant correlation (*P* = 0.004) was found between the degree of BLA infection and the PTSD-like score. *P < 0.05, **P < 0.01

To assess causality between reduced amygdala GILZ levels and the increased susceptibility to PTSD-like behavior, we mimicked the stress-induced GILZ downregulation with KD GILZ levels using short hairpin (sh) RNA expressing lentiviruses, delivered specifically and bilaterally to the basolateral amygdala (BLA) of adult naïve mice. GILZ was previously shown to be exclusively expressed in neurons^48^ and immunohistochemistry using GILZ-specific antibodies demonstrated high endogenous levels of GILZ protein in the BLA (Fig. 4a). Mice injected bilaterally with shGILZ or scrambled control-expressing lentiviruses were then assessed for PTSD-like behaviors without being exposed to the PTSD induction protocol.

Three different shRNA target sequences from the open reading frame of the mouse GILZ gene, driven by the H1 promoter and carrying a GFP reporter (Fig. 4b), were compared for GILZ KD efficiency along with a scrambled control, in amygdala AR-5 cells. A Kruskal-Wallis H test indicated a statistically significant difference in GILZ mRNA levels between the different viruses [*Χ*^2^_(3)_ = 11.00; *P* = 0.012] when normalized to GFP mRNA. Post hoc comparisons indicated that the GILZ mRNA levels of the GILZ 70–89 virus were significantly lower than those of the control virus (CV) (*P* = 0.041), the GILZ 654–672 virus (*P* = 0.041) and GILZ 386–405 virus (*P* = 0.041); all other pair-wise comparisons were not significant (Fig. 4c).

To further illustrate the efficiency of the shGILZ (70–89) in vivo, shGILZ virus or a scrambled control were injected into the BLA of male mice. Double-immunostaining using anti-GFP (green) and anti-GILZ (red) antibodies indicated that GILZ levels were reduced in mice injected with shGILZ (70–89) (Fig. 4d, left panel) compared to mice injected with scrambled control (Fig. 4d, right panel).

To assess the effect of BLA-GILZ KD on the increased susceptibility to develop PTSD-like behavior following PTSD induction exposure, 20 wild type male mice were injected with either shGILZ (70–89) or CV. Following a recovery period, all injected mice were tested in the behavioral battery previously used for subcategorization of PTSD-like mice after PTSD induction, as illustrated by the timeline in Fig. 4e. Immunohistochemistry confirmed the site of injection and one shGILZ (70–89) mouse injected off-target was removed. Comparing the distribution of mice categorized as displaying a PTSD-like phenotype across the CV and GILZ KD groups indicated that KD of BLA GILZ levels significantly augmented the symptoms of PTSD-like behavior (Supplementary Fig. 7). 67% of BLA GILZ KD injected mice (six out of nine) were categorized as PTSD-like, whereas only 10% (one out of 10) of the CV mice were categorized as such [*Χ*^2^ Continuity Correction _(1)_ = 4.328; *P* = 0.018; *φ* = 0.587; *P* = 0.011] (Fig. 4f). Furthermore, PTSD–like behaviors score correlated with the degree of the viral KD of GILZ (*P* = 0.004; r = 0.837, *n* = 8), i.e., the greater the KD, the more pronounced the PTSD-like behaviors were (Fig. 4g).

## Discussion

Male PTSD patients have reduced circulating blood GILZ mRNA levels and increased methylation, which significantly correlated with the number of lifetime traumatic events experienced from childhood to adulthood. This suggests a potential regulatory role for GILZ gene methylation in response to accumulating stressful or traumatic event exposures in males only. In mice, _CRF-induced_PNS, an early “stressor” increased the likelihood of the offspring to exhibit PTSD-like behaviors following PTSD induction in adulthood, a second ‘stressor’, and this was associated with a reduction in amygdalar GILZ mRNA levels in males and not females. GILZ expression was unique among all GR pathway-related markers analyzed in the amygdala, as its levels were reduced according to the number of stress exposures, in concurrence with the human data. This is the first study to also show sex-specific differences in GILZ, in that males with one X chromosome were more susceptible to changes in GILZ mRNA than females with two after each stressor exposure. This is also the first study to show changes in GILZ epigenetic regulation, in which there is an increase in methylation after both types of stressors in humans and mice. Furthermore, the current study uniquely demonstrated that KD of GILZ in the amygdala, in a manner that mimics the double exposure to traumatic stressors, induced reduction in GILZ levels sufficient to produce a robust increase in PTSD-like behaviors in mice.

Interestingly, GILZ KD using shRNA affects dendritic spine quality in hippocampal and cortical primary culture^48^, which may have far reaching implications for connectivity in the brain, changes in hippocampal volume in PTSD^49^ and response to psychiatric treatments^50^. Van Zuiden et al.^17^ reported elevated GILZ mRNA levels in the blood of soldiers six months after return from combat. In contrast, we report a reduction in GILZ mRNA in a cohort exposed to multiple traumatic events from early life to adulthood and assessed years later^26^. Yachi et al.^23^ demonstrated elevations in the hippocampus and medial prefrontal cortex but not in the amygdala.

In the human literature, levels of GILZ have also been shown in rheumatoid arthritis^19,51^, asthma^19^, and multiple sclerosis^52^. Peripheral effects of reduced GILZ on increases in inflammation may help explain PTSD and its high comorbidity (up to 19%) with autoimmune diseases^53^, such as higher levels of circulating IL-6, T-cell lymphocytes, increased prevalence of rheumatoid arthritis, psoriasis, insulin-dependent diabetes, thyroid disease, inflammatory bowel disease, multiple sclerosis, and lupus^10^.

The use of _CRF-induced_PNS and trauma exposed groups of mice, allowed us to explore the individual and synergistic effects of the in utero and adult challenges on GR-related markers and increasing susceptibility to PTSD. Widely-used models of PNS cause robust changes in offspring^54,55^, however these alterations are mediated by maternal corticosterone often due to physical handling and psychological stress. The inducible genetic model used to introduce PNS in late gestation in the current study controls for these confounding factors. Strikingly, late gestation manipulation of CRF had effects on GR pathway mRNA levels.

Consistent with critique in the literature (3; 16), changes in amygdalar GR, FKBP5 and CRF mRNA levels were not sensitive to the interactive effects of prenatal stress and trauma in adulthood. Only amygdalar mRNA GILZ levels were altered by _CRF-induced_PNS and PTSD induction in adulthood in a manner that corresponds with the findings in humans that associated GILZ levels with the trauma inventory in males. Further research should examine the effect on sex, gene expression and methylation in other PTSD-related or immune related genes.

The downstream effects in female PTSD, specifically related to immune function, may be induced independently of GILZ. While GILZ does have estrogen responsive elements, estrogen regulation may be activated by alternative behavioral changes or biological pathways. Incidentally, increases in the NFκB pathway also occur in females with PTSD and a history of childhood abuse. Therefore, while inflammatory pathways may also be induced in females, GILZ dependent signaling may be X-linked. Activation of the pro-inflammatory pathway in females could be GILZ-independent as either glucocorticoids or noradrenergic pathways can activate NFκB^56,57^.

Furthermore, our analyses indicated that of all the GR pathway-related markers examined in this study, changes in amygdalar mRNA GILZ levels in response to prenatal and adulthood stressors explained the highest percentage of variance. One limitation of this study maybe that the behavioral tests are considered stressful and therefore may affect GILZ mRNA levels. The only difference between the groups were age and exposure to anxiety tests. However, this may also strengthen the idea that the augmented responsiveness of GILZ occurs after each in male mice as reflected in human males. This data suggest that GILZ could be regarded as “stress quantifier” affected by each and every challenging occurrence throughout life.

Collectively, these findings suggest GILZ as a key element in predisposing males to develop PTSD. GILZ levels may serve as a tool for detecting individuals at-risk for PTSD due to a history of stressful challenges, who would benefit from an intervention to prevent any future exposure. A deeper understanding of the mechanism in females, which may be regulated by other factors, such as severity or intensity, and not stress history would further advance diagnostic use of GILZ in PTSD prediction and prevention of disability from the disease.

## Supplementary information

Supplemental materials
