# Supplementary material for: Glucocorticoid-induced leucine zipper “quantifies” stressors and increases male susceptibility to PTSD
Source: Transl Psychiatry. 2019 Jul 25;9:178. doi: 10.1038/s41398-019-0509-3 (PMC6658561; doi:10.1038/s41398-019-0509-3)
Supplement: Supplementary file 1 — Supplemental materials [file 41398_2019_509_MOESM1_ESM.docx]

Supplementary Materials and Methods

**The Grady Trauma Cohort excerpt as described in Mehta et al 2013**^1^

**Modified PTSD Symptom Scale**

The modified PTSD Symptom Scale (PSS) is a psychometrically valid 17-item self-report scale assessing PTSD symptomatology over the prior 2 weeks. Consistent with prior literature, we summed the PSS frequency items (0 indicates not at all to 3 indicates ≥5 times a week) to obtain a continuous measure of PTSD symptom severity ranging from 0 to 51. For this sample, the PSS frequency items had standardized α=.90 (mean [SD], 13.81 [11.96]). No clearly established PSS cutoff score for PTSD diagnosis has been established; however, *DSM-IV* criteria for PTSD can be applied to PSS frequency items to create a proxy variable for PTSD diagnostic status.

**Clinician Administered PTSD Scale**

The Clinician Administered PTSD Scale (CAPS) was also administered to a subset of 240 participants within 2 to 6 weeks after completing the screening assessment. We found a significant difference (*F*_1,239_=56.55, *P* < .001) between average PSS score (mean [SD], 18.20 [12.82]) for participants positive for current PTSD based on the CAPS (applying *DSM-IV* decision rules with a symptom considered as present with a CAPS frequency score of ≥1 and intensity score of ≥2) compared with those participants not meeting current CAPS PTSD criteria (mean [SD], 7.51 [9.23]). In addition, 70% of those participants identified as PTSD positive by using the PSS-based proxy variable were also positive for current PTSD at the time of CAPS administration (the criterion A traumatic experiences used for CAPS diagnosis was not necessarily the same one used for obtaining PSS data). Study participants were asked to respond to the PSS items based on the trauma exposure (inclusive of child physical and sexual abuse and other life trauma exposure) that they believed had impacted them the most.

**Traumatic Events Inventory**

The traumatic events inventory (TEI) assesses lifetime history of trauma exposure and is our primary measure of both child abuse and non–child abuse trauma. The TEI assesses past experience and frequency of 13 separate types of traumatic events as well as feelings of terror, horror, and helplessness with such events.

For the measure of child abuse, 2 of the TEI questions assessed physical abuse and sexual abuse occurring before age 14 years. Based on these questions, 17.6% of the sample reported a history of childhood physical abuse and 18.8% reported a history of childhood sexual abuse. With these data, we created a 3-level categorical variable reflecting number of types of child abuse: no child abuse (70.5% of sample), 1 type of either physical or sexual abuse (22.7%), or 2 types of both physical and sexual abuse (6.8%).

**Statistical Analysis.**

Gene-expression data.

Raw microarray scan files from Illumina HT-12 v3.0 arrays (Illumina) were exported using the Illumina Beadstudio program and loaded into R for downstream analysis ([www.R-project.org](http://www.r-project.org/)). Evaluation of the different microarray steps was done using the Illumina internal controls. Samples which were >5% SD were excluded. The data were transformed and normalized using the variance stabilizing normalization^2^. A total of 15,877 probes passing the filter criteria of Illumina probe detection *P* value of <0.01 in 5% of the individuals were used for subsequent analysis. To correct for confounding as a result of batch effects, the data were normalized using an empirical Bayes method for batch correction^3^. Reproducibility of the gene-expression data were assessed using six pairs of technical replicates, yielding average Pearson correlations of 0.996. General linear models were constructed by regressing the gene-expression profiles against the PTSD group status and adjusting for sex, age, ethnicity, substance abuse, and treatment. The significance of association was estimated by two-tailed *P* values using the ANOVA *F* test. Results were corrected for multiple testing by 10,000 permutations using the permutation of regressor residuals test (<http://cran.r-project.org/web/packages/glmperm/index.html>). Briefly, the general linear models for each transcript were built as described above and the residuals of the regressions were permuted 10,000 times for each transcript using the shuffle-*Z* method to obtain the empirical *P* values corrected for multiple testing as described previously^4^

DNA methylation data.

Raw methylation Beta values from the HumanMethylation 450k BeadChip (Illumina) were determined via the Illumina Beadstudio program and loaded into R. Internal Illumina controls were used to assess the quality of staining, extension, hybridization, bisulfite conversion, and specificity. Samples with probe detection call rates <90% and those with an average intensity value of either <50% of the experiment-wide sample mean or <2,000 arbitrary units (AU) were excluded from further analysis, allowing 163 samples for subsequent analysis. Unsupervised hierarchical clustering was performed to identify extreme outliers and global trends in methylation. One sample of male DNA was included on each BeadChip as a technical control throughout the experiment and assessed for reproducibility, with average Pearson correlation coefficient of 0.993 across all replicates. Signals from methylated (M) and unmethylated (U) bead types were used to calculate a beta value as β = M/(U + M). Hybridization and chip batch effects were accounted for using an empirical Bayes method^3^. The samples were quantile normalized and peak-corrected using the IMA package functions in R^5,6^. Hybridization and chip batch effects were accounted for using an empirical Bayes method^3^. Methylation differences were calculated using generalized linear models in R by regressing the β-values against the PTSD group and adjusting for age, sex, ethnicity, and substance abuse. Results were corrected for multiple testing by 10,000 permutations using the permutation of regressor residuals test (<http://cran.r-project.org/web/packages/glmperm/index.html>).

**Experiment 1:** **CRF-induced PNS confirmation**

**Inducible over-expression of CRF in the choroid plexus using the Tet-On system**

In order to achieve stability of our inserted gene on the one hand, and inducibility and reversibility of its expression on the other hand, we genetically targeted the choroid plexus tissue and used the bacterial Tet-On transcriptional regulation system^7^ as previously described^8,9^. The system is composed of two complimentary lentiviral vectors. The ‘Effector’ construct consists of a choroid plexus-specific promoter that drives the expression of reverse tetracycline trans activator (rtTA) protein and the reporter green fluorescent protein (GFP). The ‘Target’ construct includes the tetracycline-responsive element (TRE) DNA sequence, upstream to the nucleotide coding sequence of the requested gene of interest, followed by the reporter red fluorescent protein (RFP). Transcription initiation of the gene of interest and the RFP is mediated only in the presence of the inducer, Doxycycline (Dox). Doxycycline is the inducer of choice for our purposes as it has been demonstrated to cross the blood-brain barrier ^10,11^.

### *Production of lentiviral vectors*

### Choroid plexus-specific Tet-On lentiviral vectors were constructed as described previously^9–11^. Recombinant lentiviruses were produced by transient transfection in HEK293T cells, as described previously^8^. Briefly, infectious lentiviruses were harvested at 48 and 72 h following transfection, filtered through 0.45μm-pore cellulose acetate filters, concentrated by ultracentrifugation, re-dissolved in sterile HBSS, aliquoted and stored at -80°C. Vector concentrations were analyzed using eGFP fluorescence in HEK293T cells infected with serial dilutions of the recombinant lentivirus. Viral constructs were kindly provided by Dr. Inder Verma, The Salk Institute for Biological Studies, La Jolla, CA.

**Experiment 2:**

***Application of inclusion criterion for PTSD-like versus resilient mice***

Mice who underwent five behavioral tests: % risk assessment, reaction time to peak startle response , pre-pulse inhibition, marble burying and total light activity, were subcategorized as “PTSD-like” and “Resilient” mice based on their behavioral results.

***Dark/light transfer test***

The apparatus for the light/dark transfer test (TSE systems) consists of a Plexiglass box divided by a partition into two areas; one dark (14cm x 27cm x 26cm) the second brightly illuminated (30cm x 27cm x 26 cm, 700 lux). These areas are connected by a sliding door located at the floor level in the center of the partition. Mice were placed in the dark area and the connecting door was opened to initiate a 5 min test session. The animal’s movements were recorded and scored using a camera and automated software (Videomot2, TSE systems). Time spent in the light arena, number of visits to the light arena and total distance traveled in the light arena were measured.

***Risk assessment***

An additional arena was programmed into the software at the opening of the lit arena and extending 3cm into the light arena and along the 6cm opening. Time spent in the risk assessment area and number of visits to the risk assessment area was measured. Percent risk assessment time was calculated as the amount of time spent in the risk assessment arena as a percentage of total time spent in the lit arena outside of the risk assessment zone.

***Startle and pre-pulse inhibition***

Startle response (TSE Systems) protocol was adapted from Neufeld-Cohen 2010 et al. Briefly, mice were placed in a small plexiglass and wire mesh cage on top of a vibration-sensitive platform in a sound-attenuated, ventilated chamber. A high-precision sensor, integrated into the measuring platform, detected movement. Two-high frequency loudspeakers inside the chamber produced all the audio stimuli. The ASR session began with five minute acclimation to white background noise [65db(A)] maintained through the whole session. 32 startle stimuli [120db(A), 40ms duration with a randomly varying ITI of 12-30ms] were presented interspersed with an additional 40 startle stimuli randomly preceded by pre-pulses of either 74db(A) [40ms], 78db(A) [40ms] and 82 db(A)[40ms]. Latency to peak startle amplitude was also measured both in response to startle stimuli and in response to startle stimuli preceded by pre-pulses.

***Homecage locomotion***

Homecage locomotion was assessed using the InfraMot system (TSE Systems). Mice were housed individually for 72 hours in which the first 24h were considered habituation to the individual housing conditions. Measurements of general locomotion consisted of two light and two dark cycles in the last 48 hours collected at 10 min intervals.

***Marble burying***

Mice were placed in a compartment illuminated by 10 lux with dimensions (30 × 27 × 26 cm) containing 5 cm of autoclaved bedding with 20 marbles centrally arranged 4 by 5. Mice were then filmed for 30 minutes. Videos were scored by counting the number of unburied marbles after 25 minutes.

**PTSD-like categorization:**

For total light activity, for example, the results were sorted from highest to lowest with the highest light activity were given 1 point. The same sorting was done for marble burying, in which results were sorted from highest to lowest and the top 20% were assigned 1 point. In the case of latency to peak startle amplitude, % pre-pulse inhibition and % risk assessment, results were sorted from lowest to highest, as the extreme behavior is reflected in less % risk assessment and shorter latency to peak startle amplitude and less % ppi. In each of these three tests, 20% who had the lowest results were given 3 points for % risk assessment, 3 points for shortest latency to peak startle amplitude , and 2 points for % ppi. Points for each test were determined by factor analysis in which tests were clustered in three separate groups: (1) latency to peak startle amplitude and %risk assessment, (2) %ppi, and (3) marble burying and total light activity.

The points per animal were tallied. Mice who had totals of 5 or more points were termed “PTSD-like.” Only mice that had zero points were termed “Resilient.” The threshold of 5 was set in order to obtain between as close to 20% of “PTSD-like” mice in the population. Overall, 20% of mice were judged to be “PTSD-like” and 20% to be “Resilient.”

***real-time PCR***

Reaction protocols had the following format: 10 min at 95°C for enzyme activation followed by 40 cycles of 15 s at 94°C and 60 s at 60°C. Melting curve analysis checked the specificity of the amplification products. All reactions contained the same amount of cDNA, 10 μl master mix and 250 nM primers to a final volume of 20 μl.

**DNA extraction**

Briefly, on day 1, 200 ul lysis buffer and proteinase K (1:200) was added to each sample then left overnight in an oven at 55-60°C. On day 2, samples were vortexed gently until the pellet dissolved. Samples were then centrifuged for 5min. at 14,000 rpm/rcf, room temperature. Supernatant was then transferred from the lysate into new tubes. 300 ul cold Isopropanol was added then tubes were mixed by inversion 10-20 times. Then samples were stored for 30 min. in -20°C and centrifuge the samples for 15min. with 16000 rcf/14000rpm, 4°C. The supernatant was discarded and dried out the pellet using vacuum. Tubes were left opened in the oven 55-60°C for 5 min and when pellets change from white to clear 30 ul (first 15 ul and wash the tube walls and then add 15 ul) of DDW + RNase (5ul RNase in 1ml DDW) was added. Samples were then put back into 55 °C for 5-10 minutes, and after another up & down with the pipette, stored at -20°C.

**Experimental 3:**

***Open field (OF) test:*** The open-field apparatus consisted of a white Plexiglas box (50x50x40cm) lightened in 120lux. Each mouse was placed in the corner of the apparatus to initiate a 10-minute test session. Time spent in the center, distance traveled in the center, number of visits to the center, latency to visit the center and total distance traveled were quantified using a camera mounted above the apparatus and analyzed by TSE software VideoMot2 (TSE Systems, GmbH, Bad Homburg, Germany).

***Elevated Plus Maze (EPM):*** The test apparatus comprises a central part (5x5 cm) two opposing open arms (30.5x5 cm) and two opposing closed arms (30.5x5x15 cm). The apparatus was elevated at a height of 53.5 cm and the open arms were illuminated with 6-9lux. Mice were placed in the center, facing an open arm to initiate a 5-min session test. Time spent in the open arms, percent visits to open arms and latency entering the open arms were quantified using a camera mounted above the apparatus and analyzed by TSE software VideoMot2.

***Dark/light transfer test***

The apparatus for the light/dark transfer test (TSE systems) consists of a Plexiglass box divided by a partition into two areas; one dark (14cm x 27cm x 26cm) the second brightly illuminated (30cm x 27cm x 26 cm, 700 lux). These areas are connected by a sliding door located at the floor level in the center of the partition. Mice were placed in the dark area and the connecting door was opened to initiate a 5 min test session. The animal’s movements were recorded and scored using a camera and automated software (Videomot2, TSE systems). Time spent in the light arena, number of visits to the light arena and total distance traveled in the light arena were measured.

Supplementary Figure 1

Supplementary Figure 1. GILZ mRNA level is modified in blood of human PTSD patients in correlation with clinician administered and self-report scales of symptomology. (a) Significant negative correlation was found between CAPS score and GILZ mRNA levels (b) in addition One Cpg (cg10982861 - located in gene body/ N-shore of the CpG island) is significantly associated with CAPS (P=0.00941, r for CAPs = 0.0770) and with (c) PSS total (P=0.0488, r = 0.0712)

Supplementary Figure 2

Supplementary Figure 2 **CRF-inducedPNS affects length of gestation but no other physiological parameters.**  (**a**) No differences in maternal behavior in postpartum week 1 (b) or week 3 were observed. (**c**) Two-way ANOVA for Groups (Control/ _CRF-induced_PNS), Age (post-natal day, PND) and their interaction indicated no differences in body weight gain between offspring of the control and _CRF-induced_PNS group; *P* = 0.010. However, these differences were restricted to PNDs 32 and 41 [t_(24)_=3.902; *P* = 0.002; t_(24)_=4.294; *P* = 0.000] and disappeared at PNDs 51 and 65 [t_(24)_=1.680; *P* = 0.106; t_(24)_=1.464; *P* = 0.159].

Supplementary Table 1

|  | Control Resilient | Control PTSD-like | _CRF-induced_PNS Resilient | _CRF-induced_PNS PTSD-like |
| --- | --- | --- | --- | --- |
| Latency to peak startle amplitude (ms) | 13.28±1.36 | 9.51±0.62 | 11.62±0.50^A^ | 9.4±0.48^A^ |
| % Risk Assessment | 79.60±28.96 | 43.68±11.09 | 136.61±36.31^B^ | 34.31±7.37^B^ |
| % Marble Burying | 73.21±4.37 | 54.44±10.94 | 54.78±5.99 | 63.50±6.41 |
| % Pre-pulse Inhibition | 57.97±8.31^C^ | 29.86±14.40 ^C^ | 62.77±5.42^D^ | 38.96±8.43 ^D^ |
| Total Light Activity (AU) | 6891.71±764.44 | 7623.75±1011.13 | 6467.86±512.06 | 7322.16±836.42 |

Supplmentary Table 1. Behavioral Phenotyping of Resilient and PTSD-like behavior in _CRF-induced_PNS and controls after trauma exposure.

Control Resilient = mice not exposed to _CRF-induced_PNS and were classified as ‘Resilient’ after adult trauma exposure; Control PTSD-like = mice not exposed to _CRF-induced_PNS and were classified as,’PTSD-like’ after adult trauma exposure; _CRF-induced_PNS Resilient = mice exposed to _CRF-induced_PNS and were classified as ‘Resilient’ after adult trauma exposure; _CRF-induced_PNS PTSD-like = mice exposed to _CRF-induced_PNS and were classified as ‘PTSD-like’ after adult trauma exposure. (^A^) Latency to peak startle amplitude had a significant main effect with significant differences between _CRF-induced_PNS Resilient versus _CRF-induced_PNS PTSD-like mice (*P* = 0.014). (**^B^**) % risk assessment had a significant main effect of symptoms, with a significant difference between _CRF-induced_PNS Resilient and  _CRF-induced_PNS PTSD-like mice (*P* = 0.005). The % marbles buried had no significant main effect. (**^C^**) The % pre-pulse inhibition (PPI) had a significant main effect of symptoms with tendency toward a difference between Control Resilient versus Control PTSD-like (*P* = 0.083). (**^D^**) The % PPI significant difference between  _CRF-induced_PNS Resilient versus  _CRF-induced_PNS PTSD-like mice (*P* = 0.005). Total light activity had no significant main effect between groups. Data are mean ± SEM.

Supplementary Figure 3

**Supplementary Figure 3.** _CRF-induced_PNS **male mice with no PTSD induction in adulthood show no anxiety-like phenotype. (a)** Schema of tests performed on male mice exposed to _CRF-induced_PNS with no adult trauma exposure and their controls. No significant differences in anxiety-like behaviors were observed between the groups in the open field (**b**) and the dark/light transfer (**c**) tests. (**d**) There were no significant differences in anxiety-like behavior, assessed by the elevated plus maze test, between male mice exposed to _CRF-induced_PNS or control. Data are mean ± SEM.

Supplementary Figure 4

**Supplementary Figure 4.** _CRF-induced_PNS **female mice with no PTSD induction in adulthood show no anxiety-like phenotype**  (a) There were no significant differences in open field in females controls versus those exposed to late gestation dox. (b) In the dark/light transfer test there no difference in total time in light, and in visit, and a significant difference in the total distance covered by the mice exposed to _CRF-induced_PNS (P=0.02). (c) There were no difference between the groups in the elevated plus maze test. (n= 11-14)

Data are mean ± SEM.

Supplementary Figure 5

**Supplementary Figure 5. Female PTSD induction and behavioral phenotyping** (a) –(e) Female offspring showed no behavioral differences between control and CRF-indcued PNS . (n=11-13).

Supplementary Figure 6

**Supplementary Figure 6. Comparison of Male and Female GILZ mRNA levels after CRF-induced PNS with no PTSD induction.** There is a significant main effect of sex on GILZ mRNA levels with CRF-induced PNS alone (P=0.000). In addition, effect of CRF-induced PNS has a very strong tendency toward significance (P=0.051), meaning that CRF-induced PNS has more of an impact on males than females. Male mRNA levels are lower than females. Control male and female levels do not different significantly however.

Supplementary Figure 7

Supplementary Figure 7. PTSD-like battery in BLA GILZ KD versus control (**a**) Latency to peak startle amplitude (P= 0.003) (**b**) % risk assessment, (P=0.053) (**c**) Marbles buried, NS (**d**) % pre-pulse inhibition(P=0.03) and (**e**) Total light activity, NS. Data are mean ± SEM, student’s t-test.

1 Mehta D, Klengel T, Conneely KN, Smith AK, Altmann A, Pace TW *et al.* Childhood maltreatment is associated with distinct genomic and epigenetic profiles in posttraumatic stress disorder. *Proc Natl Acad Sci U S A* 2013; **110**: 8302–7.

2 Huber W, von Heydebreck A, Sültmann H, Poustka A, Vingron M. Variance stabilization applied to microarray data calibration and to the quantification of differential expression. *Bioinformatics* 2002; **18 Suppl 1**: S96-104.

3 Johnson WE, Li C, Rabinovic A. Adjusting batch effects in microarray expression data using empirical Bayes methods. *Biostatistics* 2007; **8**: 118–127.

4 Mehta D, Gonik M, Klengel T, Rex-Haffner M, Menke A, Rubel J *et al.* Using Polymorphisms in FKBP5 to Define Biologically Distinct Subtypes of Posttraumatic Stress Disorder. *Arch Gen Psychiatry* 2011; **68**: 901.

5 Dedeurwaerder S, Defrance M, Calonne E, Denis H, Sotiriou C, Fuks F. Evaluation of the Infinium Methylation 450K technology. *Epigenomics* 2011; **3**: 771–784.

6 Wang D, Yan L, Hu Q, Sucheston LE, Higgins MJ, Ambrosone CB *et al.* IMA: an R package for high-throughput analysis of Illumina’s 450K Infinium methylation data. *Bioinformatics* 2012; **28**: 729–730.

7 Gossen M, Bujard H. Tight control of gene expression in mammalian cells by tetracycline-responsive promoters. *Proc Natl Acad Sci U S A* 1992; **89**: 5547–51.

8 Naldini L, Blömer U, Gage FH, Trono D, Verma IM. Efficient transfer, integration, and sustained long-term expression of the transgene in adult rat brains injected with a lentiviral vector. *Proc Natl Acad Sci U S A* 1996; **93**: 11382–8.

9 Gossen M, Freundlieb S, Bender G, Müller G, Hillen W, Bujard H. Transcriptional activation by tetracyclines in mammalian cells. *Science* 1995; **268**: 1766–9.

10 Regev L, Ezrielev E, Gershon E, Gil S, Chen A. Genetic approach for intracerebroventricular delivery. *Proc Natl Acad Sci U S A* 2010; **107**: 4424–9.

11 Nau R, Sörgel F, Eiffert H. Penetration of drugs through the blood-cerebrospinal fluid/blood-brain barrier for treatment of central nervous system infections. *Clin Microbiol Rev* 2010; **23**: 858–83.
